# London Letter

**Published:** 1891-08

**Authors:** T. M. McIntosh

**Affiliations:** Thomasville, Ga.; London, England


### LONDON LETTER.

LONDON, ENGLAND, May, 1891.

*Editors Atlanta Medical and Surgical Journal:*

I left New York, April 30th, on the Augusta Victoria, with a first cabin passenger list of 200 people. It may be that a few notes by the way will be of interest to you.

Living in Thomasville, where so many come each year, from colder climates, either for pulmonary or other diseases of the respiratory mucous membranes, or simply to escape the rigors of a northern winter, and who coop themselves so closely (except the very brightest and the warmer days) in a warm, close and superheated hotel, and who avoid a draught of air, or a wind, and growl and stay in doors on a day the least cloudy or damp, the freedom with which the deck was used as a promenade, sitting or asleep, where it was damp—even wet, warm or cold, cloudy or fair, a rushing wind or a gentle breeze, day or night, fixed my attention very strongly. But it only made me more determined to make my patients with all forms of diseases of the respiratory mucous membranes, and other chronic ailments too, for that matter, follow more fully my advice to remain much in the open air. It is this prescription that has been most difficult for me to have carried out, because of the general and firm belief, that a wind or an atmosphere damp, as upon a rainy or cloudy day, was of great harm and would be the cause of "catching cold."

In the eight days of voyage, the neurasthenic became more composed, the dyspeptic gained appetite and digestion, and some with slight cough lost it entirely.

This is all attributed to the sea air, and there is, perhaps, some difference in effects upon the system between the atmosphere of the sea and land, but it would seem that if the same life in the open air was led on land as on sea, the same good would follow. Invalids on sea, and at many watering places, improve not because of the mineral water or the sea air, but because of the more healthful manner of living.

In the treatment of pulmonary diseases, Dr. Trachenix, of Saranac Lake, N. Y., has his patient to remain in the open air so long each day, no matter what the weather. This seems to be the most important part of the treatment for this terrible disease, for there is no therapeutic agent more than slightly auxiliary to other treatment. The questions of altitude, humidity and temperature have not so important a bearing as a *pure* atmosphere. Of course, a moderately high temperature is better, is more comfortable, and it *invites* to outdoor sittings and adds desire and pleasure, which would not accompany in a cold and cloudy climate. Certainly, there is no need to say that one should be well wrapped and keep warm, as they do on the sea.

While touching here, incidentally, climate, the most important agent in the treatment of pulmonary consumption, one is impressed by the entire lack of the use or even mention of Koch's lymph, here in Edinboro The first operation that I saw in London, was cutaneous lupus, by Mr. Clutten, of St. Thomas Hospital, who thoroughly scraped it in the usual way. There were other operations for tuberculous external manifestations, and Koch was not mentioned. In the case of a tuberculous or scrofulous child, an abscess in the lumbar region, due to disease in the vertebræ, was opened the second time. I say second time, because it is Mr. Clutten's method to curette out all tuberculous and inflammatory tissue after incision and evacuation of pus, disinfect thoroughly and close the wound properly. He uses no drainage tube because there will be, he thinks, more or less infection by it, with the greatest care. He reopens the abscess should pus again form, and again closes wound as before. The drainage tube in such cases by most surgeons would be used, and, it would seem, to better advantage.

Mr. Croft, of St. Thomas, resected the articular ends of all the bones of the elbow joint in tuberculous bone disease and was extremely careful in preserving all of the muscular and ligamentous attachments, by keeping the knife well against the bone. This he explained as Mannder's operation, and expected to have a fairly good arm, and would later on begin passive motion.

On this subject of passive motion, after resections, Mr. Duncan, of the Royal Infirmary, Edinboro, made a long clinical talk, in which he said that in *reality* there was no "passive motion," and he made no effort in dressing such cases to prevent motion, as the patient would make but little any way, and if so was only what would be done by the surgeon, *actively*, later on. It was merely, after all, a difference in terms in relation to the motion by patient or in the abstract, but the point was to allow motion by the patient as freely as it wished, by not having the splint adjusted with the perfect rigidity that is usual.

In Edinboro, at the Royal Infirmary, completed in 1875 at a cost of one and one-half million dollars, there is much to be seen.

Mr. Annandale operated on a large, double adherent inguinal indirect hernia. One side, Mr. McEmon's method, of thoroughly tearing all adhesion of the sac and then folding it, as it were, by suture, and stitching in firmly to the borders of the ring; the other, the sac, after being freed from attachments, was cut off, and the stump stitched to the pillars of the ring, with the same sutures used in closing external wounds. Some operators use a separate suture for the peritoneum and the abdominal parietes, but from good results from either method, we would conclude that there was no difference in their value. In a case of strangulated hernia, of five days, in my own practice, after the return of the intestines into the abdominal cavity, there was a large mass of protruding and irreducible omentum which I amputated well up to the ring and brought the cut edges together in the same manner as Mr. Annandale.

There was a good recovery. Mr. Annandale's assistant, in a case of obstruction of the bowels of seven days' duration, opened the abdomen but found no cause for the obstruction but a long, stringlike piece of peritoneum extending from an attachment over the diaphragm to the inguinal region. An ineffectual effort was made to produce a movement of the bowels by large injections of water. The result of operation I could not learn, as I left for London again the next day. Through the kindness of Dr. Battey, who gave me a letter of introduction to Dr. Simpson, I had the pleasure of accompanying the latter to a meeting of the Royal Obstetrical Society, but no paper of interest was read, though a short one by the President, Mr. Berry Hart, on a new nomenclature for presentations, based upon anatomical relations, elicited some discussion, the sense of which was that the present classification was better.

At King's College Hospital Sir Joseph Lister operated upon a case of fracture of the patella by suture with silver wire. There cannot be osseous union in this fracture, because, by muscular contraction, the planes of the fractured bone are almost at right angles to each other, and the periosteum and tissue above being put on the stretch before rupture to more than normal, when the fractured ends of the patella are approximated by any apparatus, this stretched and torn tissue drops over the fractured edge of the fragments and is a periostael and other tissue cushion between them and prevents necessarily other than ligamentous union, even though the apposition be otherwise perfect. This interposed tissue is most carefully cut away, so that only bone surfaces are in contact. This detail is important. The wire used is one-twentieth of an inch silver. A common brad awl is used for working the hole for suture, and begins about half an inch from the fracture, on the external surface of the patella, and is brought out just at the interior edge, just above the periosteum.

If the holes in the fragments should not be in perfect line, the bone above the lowest suture is picked away with the awl until it is of the same height as the other. In tying the wire a half knot is made and the ends then bent across each other as a chain link, which prevents slipping and is not too large for the proper covering by the skin. A posterior splint is used, and passive motion, when the union of bone has been complete.

All of the London surgeons do not wire fractured patella, but content themselves with only a fairly useful limb from union by ligament, thinking the risk of an inflammation of the knee joint and its results too great to justify it, notably Mr. T. Holmes.

Yet Lister makes the operation and gets his good results. Mr. Holmes does not and has imperfect limbs.

Let each surgeon and each patient decide what will be done. Much depends upon the occupation of the patient as to whether the wiring treatment is imperative or not. It is always better. In the wards was a patient with dislocation of seventh cervical vertebra, for which Mr. Lister, a week before, had operated by wiring the spinous processes together to prevent displacement which takes place after reduction. There was almost complete paraplegia, in which there had been no improvement from the operation, and a fatal termination was feared because of the injury which occurs, in such cases to the spinal cord at the time of the accident and by the pressure of the dislocated vertebræ upon it. In future cases Mr. Lister proposed to remove the laminæ of the vertebra, as well as wire the spinous processes, so that should there be slight displacement the pressure upon the spinal cord would not exist.

Every one knows that Mr. Lister has discarded the spray in his operations, and in his lecture he said it was impossible to get any wound free from bacteria, for even in the laboratory one out of five of the sterilized tubes would have bacteria, which the experimenter regarded as a good percentage. He said that the human organism had a certain power to resist the action of bacteria, and this power was the aid to the surgeon in procuring non-suppurative wounds treated by as thorough an antisepsis as possible, in the operation. Practically this is but the "vital resistance" of pre-bacteric time, and the phagocyte and its opponent, of the present date. And while it is of little importance to enter into a discussion of the comparative value of antiseptics (or their value at all) and asepsis in surgery, it would seem that Mr. Lister, in his clinical lecture, really acknowledged that thorough cleanliness was all that could be obtained in surgical wound treatment, and that antiseptics had but little to do with the good results in modern surgery, and of which Mr. Lister is the undoubted and justly acknowledged and celebrated author. In a short paper, "Cases in Surgery," which I presented to the Medical Association of Georgia, at Macon, in 1884, it was held that *perfect* cleanliness, such as would be given by an *abundant* and *thorough* use of warm water, perfect apposition and fixedness of wound surfaces, a dressing with an abundance of absorbent cotton to absorb secretion, maintain equable pressure and preserve a normal warmth of the wound, that the results would be as good as by the use of bichloride or carbolic acid solution. A short time before leaving home I amputated a leg, just below the knee of a negro injured by a railroad accident, cleansed well with soap and warm water, used no antiseptics on either sponges, ligatures or sutures, cut the ligatures close, closed the wound without drainage, dusted some iodoform over the wound, dressed with gauze and cotton, and in thirteen days redressed the wound for the first time. It had healed. There was no suppuration. The ligatures around the arteries (of silk) gave no trouble, and yet no antiseptic had ever touched them. But, as before said, so that hands, sponges, instruments and everything used in an operation be *clean*, it is of little importance, practically, whether antiseptics are of value or not, for the patient cares not whether he is cured with or without a particular agent, but the exact scientist desires to know which and what agent, or combination of agents, produces his results.

It may be of much interest to know that chloroform is almost exclusively used here and in Edinboro, and that it is simply poured in small quantities at a time upon a folded napkin or handkerchief.

Only twice have I seen ether used at all (with a modified Claven's apparatus) where a prolonged anæsthesia was necessary. To most American surgeons, at least, this may seem rather an unwarranted risk in the choice of an anæsthetic, after the very able and interesting paper of Dr. Wood, of Philadelphia, read before the International Congress, in Berlin, in which he set forth as the result of experiment the greater effect of chloroform than ether in depressing and stopping the action of the heart, to lessen which he advised the administration of digitalis hypodermically just before beginning the anæsthetic (sometimes also, nux vomica) as a cordial, tonic or stimulant.

I will remain a few days in Paris, *en route* to Berlin and Vienna, and may send you a few notes from that place.

T. M. McINTOSH, M. D.,
*of Thomasville, Ga.*

---

TRANSMISSIBILITY OF SYPHILIS.—As published in his magnificent atlas of *Venereal and Skin Diseases*, Prof. Morrow's conclusions in reference to the hereditary transmission of syphilis are :

1. A syphilitic man may beget a syphilitic child, the mother remaining exempt from all physical signs of the disease ; the transmissive power of the father, is, however, comparatively restricted.

2. A syphilitic woman may bring forth a syphilitic child, the father being perfectly healthy; the transmissive power of the mother is much more potent and pronounced, and of longer duration than that of the father. When both parents are syphilitic, or the mother alone, and the disease recently acquired, the infection of the fœtus is almost inevitable; the more recent the syphilis the greater the probability of infection, and the graver the manifestation in the offspring.

3. While hereditary transmission is more certain when the parental syphilis is in full activity of manifestation, it may also be effected during a period of latency, when no active symptoms are present.

4. Both parents may be healthy at the time of procreation, and the mother may contract syphilis during her pregnancy, and infect her child *in utero*. Contamination of the fœtus during pregnancy is not probable if the maternal infection takes place after the seventh month of pregnancy.—*Weekly Med. Review.*

